# The Principles and Practice of Medicine, Founded on the Most Extensive Experience in Public Hospitals and Private Practice, and Developed in a Course of Lectures Delivered at University College, London

**Published:** 1839

**Authors:** 


### THE PRINCIPLES AND PRACTICE OF MEDICINE, FOUNDED ON THE MOST EXTENSIVE EXPERIENCE IN PUBLIC HOSPITALS AND PRIVATE PRACTICE, AND DEVELOPED IN A COURSE OF LECTURES DELIVERED AT UNIVERSITY COLLEGE, LONDON. By *John Elliotson,* M.D. F.R.S. With Notes and Illustrations by *Nathaniel Rogers,* M.D. &c. Joseph Butler, London, 1839. Octavo, pp. 1088.

WE confess it was with some degree of apprehension lest the untoward subject mesmerism should obtrude upon our notice, that we first turned over the pages of this work. We are glad, however, to find no allusion to that cunningly devised fable; and feel persuaded that Dr. Elliotson himself, sooner or later, will by no means regret the omission. The records of the practice of mesmerism had been sorry company for the goodly array of sound theories, clear philosophical expositions, and judicious views here set forth. Would that such a consideration had operated to prevent the late transactions at University College! And it ought to have done so; for if mesmerism were "as gospel true," its Apostles could never reasonably claim admission for it into the temple of science, until it had been *proved* not merely as not a fiction, but as an assemblage of indisputable facts and phenomena demonstrative of some natural law or rational theory. Those great discoveries which, at their first promulgation, were so violently assaulted and derided, were of this character, and, even in the height of their disrepute, every man of competent intellect, who examined into them with sincere and unbiassed feeling, might have ascertained their basis upon the eternal rock of truth. Moreover these were elaborated and wrought out in the closet through many long years of patient investigation: they were not thrust into notice, nor exhibited on a stage to groups of idle and wondering spectators. Silently and calmly they withstood opposition, ridicule, and persecution; and at length, by their own innate power and worth, won their way to the eminence they now and ever will enjoy. But animal magnetism has been introduced to our notice in such harlequin garb and gesture; playing such fantastic tricks—appealing so much to the senses, and so little to the reason—striving to dazzle and amuse rather than to instruct and convince—that we are inclined to say, "thou com'st in such a questionable shape, we'll call thee '*humbug*.'"

We do not deny certain of the extraordinary phenomena ascribed to mesmerism, but are not some cases of catalepsy, chorea, trance, &c. equally extraordinary, and equally capable of being traced to an unwonted and morbid state of the nervous energy, rendering it susceptible of influences before unknown and unnoticed, especially perhaps of agencies accounted trivial in a healthy and normal condition of the system. We may instance inflamed membrane and muscle as analogous to this *status,* and particularly those cases of neuralgia in which the slightest touch will cause exquisite pain, while strong pressure has no such effect. Then we have to consider the exciting causes—the presence and close contact of the magnetiser—an individual of the opposite sex—the consciousness that his eye, his thoughts, his manipulations are directed and concentrated upon the patient's self;—the expectation of certain predicated effects, the excitation of an imposing assembly, the contagion of example, &c.: all those influences acting upon the imagination, which the circumstances suggest to the reflecting observer, and which we think quite sufficient to account for the genuine phenomena. Moreover we must not forget that the disorders of the nervous system to which females are peculiarly subject, as hysteria, chorea, &c., are *sometimes* to a considerable extent under the control of the will.* "De reliquo," it is doubtless sheer fraud and deception, as in the instance of Miss O'Key, whom we scruple not to designate a first-rate actress in her line. We need only refer to her performances at University College a few months since, when in the height of her fame, and evidently progressing in the practice of her art. If her career had not been checked, we wonder how far her impudent reliance on the gullibility of mankind would have carried her: she had already ventured on prophecying. Pity it is she had not flourished a few centuries ago, she had then vied with the Cumæan Sybil, and might have practised her vocation without fear of sceptics and revilers like ourselves. Had Greece been honoured as the land of her birth, she might have attained equal celebrity with the sages of antiquity; for, like Socrates, she has her demon or familiar, alias the *negro;* to whom she appeals for direction, and who opens to her the mysterious future. Seriously it is matter for regret as well as surprise, that a man of Dr. Elliotson's extensive acquirements and long experience should have become the dupe of an artful girl, and the victim of such a farcical delusion. At the same time we would not for a moment doubt or impugn the sincerity of his motives, and the perfect good faith of his conduct in the affair of mesmerism; and, we dare to say, he considers himself a martyr suffering like Galileo and Harvey in the cause of truth and science. We expressed surprise at this hallucination in Dr. E., for who could have supposed that he who in his "Physiology" argues so strongly against the doctrine of an immaterial principle or spirit, should so readily take up with the supposition of an ethereal vital communicable fluid, transcending all the known laws of physics, and which, if it do exist, would appear to be a kind of "via media" between matter and spirit; but more closely allied to the latter, according to the usually received ideas of a spiritual essence. But enough of Animal Magnetism, our readers will begin to say, we have had it brought before us *ad nauseam*. We will turn then to the work before us, merely congratulating the author and the public that it contains not a word about mesmerism.

This volume is inscribed in a very neat dedication to Lord Brougham, and

* Dr. E. seems quite aware of the Protean forms and marvellous phases which the nervous complaints of females are apt to exhibit. Thus at p. 738 of the work before us he says, "Very extraordinary cases independent of organic disease are to be seen in young women. Cases are continually seen of so extraordinary a nature, that you would not believe in their accuracy if you merely read them in books. I recollect very well seeing a young lady, single—for these cases almost all occur in single ladies," &c. Again, speaking of France, prior to mentioning a case, he observes, "these strange things generally occur in females," (p. 547.) We may also refer to his remarks on hysteria, (538 et seq.) and on *fidgets*, (p. 544.)

is ushered before the public by the editor, Dr. Rogers, in the following apposite terms:—

"One of the most striking circumstances which have distinguished the medical literature of recent times, is the publication of the Lectures of various eminent Professors. Till within the last few years, hospital-theatres, as well as hospital-wards, were (except to the limited number of attendent pupils) as "a spring shut up," and "a fountain sealed." It would have been of the greatest advantage to medical men, and through them to the public at large, could these class-rooms have contained the whole profession. But what the architect could not do, the printer has done. He has given the Lecturer a voice, which penetrates as far as our language is known; and the results of his study and experience have been wafted, on the winds of heaven, to every quarter of the globe. Beginning with Sir Astley Cooper, the masters of our art, in brilliant succession, have thus been enabled to pour the accumulated treasures of their knowledge at our feet.

The name of Elliotson stands too high in the estimation of the Medical Profession, to require any apology for the publication of his Lectures;—replete, as they are, with sound practical information;—the result of long and extensive experience. There can be little doubt, therefore, that the present undertaking will be hailed with satisfaction; and that a due share of support and encouragement will be awarded to the enterprising publisher, for producing this work in a style commensurate with the valuable information it contains."—*Preface.*

As we shall have occasion hereafter to advert to the praiseworthy manner in which the editor has fulfilled his office, we pass on to notice the general features and contents of the work.

In his Introduction the learned Professor points out the value and necessity of medical lectures and clinical instruction, the advantages of a museum, the superiority of hospitals and dispensaries, and the imperative need of having a hospital attached to the school where medical and surgical precepts are delivered. He makes the following very appropriate remarks on the education of the youth of our profession.

"No better education is given by parents to those children intended for medical practitioners, than to those whom they destine for trade; and at the same time when the best part of general education should begin, the youth, with all his capabilities of literary and philosophical attainment, is hurried off to mix draughts and weigh powders, the greater part of the day, for several years. The portion of time thus consumed, and the sum of money thus expended, would enable the youth of our profession to attain every acquirement of a high education. The young men who come to our schools, to prepare themselves for general practitioners are, in the majority of instances, as gentleman-like in their sentiments and manners, as intelligent, as anxious to learn everything that their instructors are disposed to teach them, as those who are sent to Oxford or Cambridge,—as young men of whatever station in society, and in the previous enjoyment of whatever advantages. I trust that ere long the five years' apprenticeship to learn the business of the dispenser, or (as that might be learned in as many months) I should rather say, the five years' apprenticeship to waste their time as shopmen, will be abolished; and instead of the present preposterous regulations, of five long years in the shop before a limb is dissected,—before a lecture on Anatomy, Chemistry, or any other subject is heard, and two years only in systematic public study,—in dissecting and attending so many lectures, and in gaining the experience afforded by the medical and surgical wards of hospital, and in reading (which previously would have been fruitless), —the youth will, through the medium of such establishments as the London

University, acquire a good classical education, a knowledge of the three great continental languages,—the French, German, and the Italian,—and no inconsiderable acquaintance with Mathematics, Physics, and Mental Philosophy. By a change of plan, and by practicable facilities, I am confident that all this may be accomplished with no great increase of expense; and the young practitioner be ready for the commencement of his active career, at the age of two or three and twenty; and no one will deny, that a given expenditure of money and of time, ought to be made to produce the greatest possible advantages. These facilities will all be amply furnished by this University; and I hope that the Apothecaries' Company will one day be better pleased that those young gentlemen who go before them, should adduce testimonials of a respectable classical and mathematical education, a capability of reading the German, French, and Italian writers in the originals, and the possession of a share of Physics and Mental Philosophy; than if these young gentlemen proved they had consumed five years, when the mind thirsts most ardently for knowledge, in drug-mixing, or such poor and desultory instruction as a private practitioner has time to bestow." 8.

"If I am told that this improvement in education will make the general practitioner equal in knowledge to the physician, I reply that this will rejoice me beyond measure. I have no desire that the importance of physicians should be maintained by the depression of the general practitioner." 9.

The Dr. is not an advocate, however, for "one faculty," and his subsequent observations will tend, we imagine, rather to neutralize the effect of the above admission on the minds of the "levellers;" since after conceding thus far, the "rock of offence" remains still the same; it is in the barrier where lies the grievance. The physician allows the general practitioner to attain the same eminence where he himself stands, but immediately he takes a stride upwards, so that the relative distance between the parties is still just the same as ever. The remarks to which we allude are these:—

"If the physician is to continue as superior to the general practitioner as he formerly was, he must advance in the same proportion; he must not presume to think of preventing the general practitioner from stepping towards him; he must also step forwards himself, to maintain his advantage. The physician whose means have allowed him to spend more time than the general practitioner in his literary and scientific education, and in the subsequent study of his profession, and has spent it industriously; and who, when in practice, and exempt from the toils of midwifery, and the other distractions which beset the general practitioner, and having not the whole of the art, but one branch only to cultivate, employs these advantages as his sense of duty must dictate,—will always acquire every reasonable advantage; and general practitioners will gladly avail themselves of his assistance, and will be proud of him as a supporter of the character of the profession." 9.

He thus expresses his deep sense of obligation to the body of general practitioners.

"When I commenced my professional career, I determined upon seeking for success by working hard; and by conducting myself as well as the infirmity of human nature would allow. I determined, however long I might wait for success, never to fawn upon and run after my superiors, nor to stoop meanly to my inferiors; never to intrigue for an advantage, nor to employ trumpery artifices for making myself known to the public. For many years I toiled; and saw most of my contemporaries, nay, of my juniors (who worked less, but were wiser in their generation) pass by me. I published work after work—edition after edition; and paper after paper was honoured with a place in the Transactions of the first Medical Society in Europe. I was physician to a large metropolitan hospital; and had attended there, and gratuitously out of doors, above twenty thousand patients. But in vain. In 1828 my profession was no more lucrative to me, was as short of my actual expenses, as it had been in 1818. At that time the *Lancet* was pleased now and then to publish a clinical lecture, delivered by me at St. Thomas's; and my practice at once doubled. The following year it published the greater part as I delivered them; and my practice doubled again. Last season the *Lancet* published them all; the *Medical Gazette* followed its example; and my practice has now doubled a third time. This astonished me; the more as my clinical lectures were generally delivered with little or no premeditation; while all that I published myself had cost me great labour, many a headache, and much midnight oil. It was through the general practitioners, in the large majority of instances, and through general practitioners, for the most part, with whom I had not the honour of any acquaintance, that the publication of those lectures accomplished my success. To the body of general practitioners, therefore, I owe a debt of gratitude. They have called me forth spontaneously from no interested motive; and I cannot exert myself too much in the education of their successors."* 10.

Having finished his "introductory remarks," our author proceeds to make some "general observations" on the laws of matter; the causes of diseases, as mechanical violence, sudden alternations of temperature, bad air, unwholesome food, &c.; the characteristics of disease; the division of diseases into functional and structural, local and general, acute and chronic, &c. He manifests a very laudable anxiety that his auditors should have a correct notion of the meaning of the phrases employed, for instance—

"You will find that diseases are also considered, by some, accordingly as they are *active* or *passive*;—that is to say, accordingly as they are attended with a degree of violence or excitement of the system, or no excitement at all. You will find some persons (indeed a large number) use the terms '*active*,' and '*passive*,' and '*acute*' and '*chronic*,' synonymously; but this is an error against which I am particularly anxious to guard you. It does not follow because a disease is acute—that is to say, exists for a short time, and then terminates—that it is necessarily attended by violent symptoms; nor does it follow, because it lasts for a length of time, that these symptoms should not be active. This is every day illustrated in the case of rheumatism. It is daily spoken of as acute or chronic, and active or passive; the term acute being used indifferently with active, and chronic with passive; so that, in the chronic form of the affection, a person would not think at first of applying such remedies as in an acute attack; taking it for granted that, as the disease is chronic, there is no violence; but that a slowness of mischief is going on, and that the remedies for the active acute state are improper. But you see cases of rheumatism every day, which have lasted for six or twelve months, or even longer, attended with all the symptoms of an acute disease;—that is, attended, if not by quickness of pulse, by heat of the parts; and if you take away blood, you find it buffed. It would,

* We think our reviewer might here have noticed a remarkable fact—namely, that the very man and the very vehicle which doubled, quadrupled, and sextupled Dr. Elliotson's practice, and consequently his fame, did, a few years afterwards, annihilate the one, and damn the other! We need scarcely allude to the exposure in Bedford Square, and the "crowner's quest" on the sayings and doings of the O'Keys. Would the reputation of a Baillie, a Cooper, or a Brodie, together with the emoluments of their practice, have risen and sunk in this rapid manner? Our readers may answer in their own thoughts!—*Editors*.

therefore, be a serious error, if you were to consider '*active*' and '*passive*,' as synonymous terms with '*acute*' and '*chronic*;' because in acute diseases, the word '*acute*' refers simply to their short duration; and in chronic diseases, the word '*chronic*' simply to their long duration; whereas the word '*active*' refers to the violent disturbance going on, in the system or part, and the word '*passive*' to slow changes,—changes not of a violent description. A *chronic* disease may be *active*; and an *acute* affection may be *passive*. A person with an acute affection may lose all his powers, and the whole functions of the body may go on in the most languid way; and therefore acute and chronic are one kind of thing, active and passive are another. An *acute* affection cannot be *chronic*, but it may be *passive*; and a *chronic* affection cannot be *acute*, but it may be *active*." 17.

"With respect to the *causes* of disease, you will find them generally divided by authors into two,—the *remote* and the *proximate*. The remote causes, again, are divided by systematic writers into two more, *predisposing* and *exciting*. The word '*cause*,' however, is in these cases used in a totally different sense. When we speak of '*remote* causes,' a very different idea presents itself to you than when we speak of '*proximate* causes;'—in fact, the cause of the disease, properly speaking, can only be remote. For example, the remote cause of fever may be bad food, and the depressing passions; and the exciting cause may be, according to some, a specific contagion. Now the remote causes of the first description are called *predisposing*; they render the body liable to become the prey of something, which has a tendency to excite the disease. The exciting cause of the disease might have had no effect, unless the body had been predisposed; and the predisposition might not have had the effect, unless the exciting cause had occurred. A circumstance, therefore, which inclines the body to become the subject of disease, is called a predisposing cause—*causa prædisponens*; that which actually excites the disease, exciting—*causa excitans*; and both together are called *causæ remotæ*. They are called remote, I presume, because they are a little distance from the disease itself; and because the proximate cause is, to all intents and purposes, close to and all but the actual disease; and, in one sense at least, it is the disease itself." 19.

"The application of this term gives rise to a great deal of confusion to the student. You would imagine that disease could be nothing more than disease; and to call the disease the proximate cause, would be thought absurd; but the reason of this I will explain. In defining any particular disease, we are obliged to take into our definition only what we observe; just as the naturalist defines any flower, or any mineralogical specimen, by merely taking what is the object of sense, and describing it with all the marks together; and thus we have what is called the definition of a disease. For example in jaundice you take the yellowness of the skin, the yellowness of the eyes, the yellowness of the nails, the whiteness of the fæces, and the high colour of the urine, together; and you say that the patient certainly has jaundice. The jaundice is not the disease, strictly speaking; but the collection of symptoms under which the patient labours, is called jaundice. The word '*disease*' is thus applied by nosologists, not to the single *cause* of all these symptoms, but to the *collection* of symptoms. Take epilepsy: a person falls down, foaming at the mouth; struggling in every limb; perfectly unconscious; and, afterwards, when he ceases to foam and struggle, he lies senseless; such a person is said to labour under epilepsy. These particular symptoms, blended together, occurring in succession, are called epilepsy. Now in Cullen's, and other methodical nosologies, you will find the disease defined according to mere symptoms;—all opinion, all cause, is excluded. You take merely the symptoms, and you call them the disease; and it is perfectly right that it should be so. But these symptoms must have a cause; there must be a cause for this disturbance in epilepsy;—there must be a cause for the bile going into the blood in jaundice, appearing in the urine, and not being able to find its way into the fæces. The cause of the epilepsy, or of the jaundice, is the circumstance that produces all these effects; and that is considered to be the 'proximate' cause. If the epilepsy arose from a piece of bone depressed upon the brain, you would say the proximate cause of the epilepsy was the spiculum of bone. If the bile were obstructed because a calculus was impacted in the hepatic duct, you would say the impaction of the calculus was the proximate cause of the jaundice. You therefore observe that the proximate cause is, in fact, the disease itself;—the actual disease from which the symptoms arise. But it is necessary you should know, that the term '*disease*' is employed by methodical nosologists, and must be so by me to signify a collection of symptoms, and not the true cause which is at the bottom of those symptoms." 20.

We cannot conceive how the cause of a disease can, without an abuse of language, be called the disease itself. Both the etymology, and common-sense use of the term disease, implies effect and not cause; and the same thing cannot be at once both cause aud effect. Let us consider the above illustration of the proximate cause of epilepsy a little more closely. A spiculum of bone may irritate the brain, and cause epilepsy; and since the spiculum of bone is the "proximate cause," which is "the disease itself;" therefore a piece of bone is a disease! And so of the impaction of a calculus in the hepatic duct as the proximate cause of jaundice. On referring to Hooper's Physician's Vade Mecum, (dedicated, we observe, to Dr. Elliotson,) the proximate causes of epilepsy and jaundice, as above explained, are enumerated among the existing causes of those affections, and the "proximate cause" of jaundice is defined to be "the absorption of bile into the sanguiferous system." While, in the introductory part of this work, "proximate cause" is said to mean "the exact condition of an organ when its structure or function is diseased," (*Vade Mecum*, p. 11.) Darwin, with more propriety, designates this "proximate cause" *proximate effect;* but he is censured by Dr. Mason Good, (Nosology, Prelim. Diss.) who observes, "by proximate cause is generally understood, the most striking or characteristic symptom of a disease; while his (Dr. Darwin's) "proximate cause" is the *remote cause*, as the phrase is commonly explained. Confusion must naturally result from such conflicting definitions, and we would abjure the use of the term "proximate cause" altogether, if it could not be employed in a less ambiguous and contradictory sense.

Instead of "predisposition" to certain diseases, our author would rather say "want of indisposition." It is manifestly inconsistent to use the term "predisposition" as a cause of diseases resulting from mechanical injuries, where the natural constitution of the animal fabric, or the condition of the part as an organic structure, is all that is previously required, in order that certain results may follow the infliction of the external violence. We do not see why instead of predisposition, the term *disposition* should not be used, which would indicate the state induced by what are often called "exciting causes," viz. stimulating and depressing agencies. We cannot quite coincide in the following sentiments, and, from a foot-note of the editor's, he appears to think with us:—

"We must not, in medicine, be very nice about the etymology of our words;—that is to say, we must employ words of a certain meaning, so as to be clearly understood; but we shall find words signifying something very different from what their etymology would lead us to suppose. Nor is this remark confined to Medicine; it applies equally to Anatomy;—and our only object should be to become understood among ourselves;—to know what ideas are meant to be conveyed, when certain expressions are used." 26.

A conventional use of terms is all very well, if it do not create discordance, as is too often the case; and it is greatly to be regretted, that coiners of technical terms, and authors in general, have not had more regard to the exact etymology of words: it would have prevented many barbarisms, much confusion, and those repeated changes and synonymes which perplex as well as tax the memory, and often mislead the understanding. Precision in language is always auxiliary to precision in ideas.

In this preliminary part of the work, and subsequently, we meet with perhaps rather too much of recapitulation; for instance, a great deal that is stated at p. 17—18, occurs again, with some modifications, at p. 24—25. This repetition is certainly often required in teaching the elements of a science; it is necessary for the tyro to have "line upon line" and "precept upon precept," but we think it superfluous in a work calculated for the use of those to whom such information must be quite familiar. A work of this value will find its way into the library of every practitioner in the kingdom.

Having considered the six non-naturals, (as they have been absurdly called,)—the temperaments,—diathesis,—the inferences to be drawn from the senses,—the surface,—the pulse,—auscultation, &c. the learned Professor proceeds to notice the method he intends to adopt in speaking of the various subjects he has to enunciate. He avows himself a decided enemy to methodical and nosological arrangements.

"Now, however useful it may be to arrange diseases slightly, for the purpose of memory, and for the purpose of general views, I think it must be confessed, that all these various *methodical nosologies* only perplex and encumber the mind. I studied the arrangement of Dr. Cullen formerly very minutely, and had great part of him at my fingers' ends; but I confess that my knowledge of it now is but of a very superficial kind, and that it was never of any use to me. The arrangements of Drs. Young and Good appear to me just as useless; and I would not, if I were to advise you honestly,—and I hope I always shall do so,—I would not advise you to plague yourselves about nosological arrangements. My own experience tells me that it is a much greater plague to recollect the arrangement, and all the hard words, than to recollect the things for which the arrangement is made. I never found it of the slightest use; any more than the barbarous jargon of the *Propria quæ maribus*, and *Quæ genus* of the Latin grammar.

The most natural mode, in my opinion, in which we can attempt to arrange diseases in our mind,—that which serves best for the purposes of recollection (for an arrangement is certainly useful; although I am not an advocate for a *methodical* one, so called) is a two-fold arrangement; first, as to the nature of affections in general,—whether they are inflammatory, structural, functional, mechanical, or parasitical (for whenever we see a case, we immediately consider what is the kind of affection); and then, secondly, as to the part in which the affection occurs. This is the arrangement which I shall follow. I shall first consider general diseases,—such as affect every or most parts of the body;—inflammation, scrofula, and various other organic diseases; and afterwards, having considered all the affections which may attack any part of the body, I shall proceed to consider those affections, and all others, whether functional, mechanical, or parasitical, as they attack the body from the head downwards—*a capite ad calcem*. I think we all make two inquiries in considering any case;—the one is the nature of the disease, and the other is the situation of it." 59.

In accordance with his plan, our author first considers general affections, as inflammation, hæmorrhage, change of structure, &c.; next universal diseases, as scurvy and chlorosis; he then treats of fevers,—intermittent, continued and remittent; and then of cutaneous diseases, among which he classes the exanthemata. He forthwith enters upon the diseases of the interior, commencing *a capite*. This method being pursued, one might naturally inquire, where are those affections classed the exact seat of which cannot positively be determined,—which attack sometimes one part and sometimes another, or which implicate distant parts? They are thus disposed of;—tetanus, chorea, neuralgia, hydrophobia, &c. are considered among diseases of the head; as also spina bifida, which is placed between convulsions and delirium tremens.

But we must allow the Doctor to explain this discrepancy, although, in so doing, he departs somewhat from his original plan. Speaking of tetanus—

"This disease may not have its source exactly in the head, but in the spinal marrow. I am, however, an enemy to all strict arrangement; and therefore I shall speak of it, and all other diseases of the spinal marrow, at the same time that I speak of those of the head. The head contains the chief part of the nervous system; and it is more convenient to speak of diseases of the nervous system at large, when speaking of the head, than to divide them, and speak of them at distant intervals." 479.

Gout is treated of subsequently to affections of the urinary organs, and is followed by rheumatism, which completes the course. We are thus, according to promise, conducted by gradual descent not only "*ad calcem*," but "*ad pollicem pedis*."

We will now give a specimen or two of the style in which the various topics are handled: but first advert for a moment to our author's fondness for physiological disquisitions which is often apparent, some may think too much so, especially when the conclusions are after all conjectural. At p. 78 et seq. all that relates to the cause of the buffiness and coagulation of the blood, as connected with the "Practice of Medicine," might be contained in one page; and at last we have the honest acknowledgment—

"I cannot ascribe the coagulation of blood either to its life or death, or to its escape of carbonic acid. I therefore beg to say, that I do not know why blood coagulates. I am not satisfied with the reasons that have been given; nor am I not prepared to advance another reason instead of them; and it is always a great point gained to know one's ignorance.

The real cause, therefore of the buffiness of inflammatory blood, certainly is not the slowness of the coagulation; but from what it proceeds, I do not know." 79.

"Nor am I not prepared," should be "nor am I prepared."

In common with Willan, Rayer, and others, Dr. Elliotson classes measles, scarlatina, small-pox, and even plague among "cutaneous diseases," the two former in company with erythema, roseola, and urticaria, as *exanthemata* or *rashes;* variola under *pustulæ*, and plague under *furunculi* or *boils*. Now, whether they be called exanthemata or not, we think the ancients were right in considering them "eruptive fevers:" they are essentially pyrectic or inflammatory—they have all the characteristics of fever, accompanied with an eruption; but we view the skin as implicated only secondarily, while the prime mischief resides far beneath the surface.

We are glad to find the doctor protesting against the necessary subdivisions, changes, and pedantic epithets of some authors. Speaking of strophulus;—

"You will find it divided, by authors, into a number of tiresome varieties; which only serve to burden the memory, and are soon forgotten when you come to practice. The great point which I would urge upon you at present is, to recollect the characters of diseases *in general;* and as to the particular *varieties,* learn them afterwards, as different cases present themselves to your notice; and then do not trouble yourself so much about the *names* of these varieties, as about remembering the fact that there *are* some varieties in the appearances of the affections; and still more (what is not enough dwelt upon by subdividers) some varieties in the condition of the *system,* and of the *part affected* in these diseases." 332.

Again, of erythema and roseola, and of lepra—

"To shew you the absurdity of separating these two diseases, I may mention that one is called 'red patches, variously figured and irregularly diffused;' and the other is called 'red patches, or diffused redness.' I am sure it is frequently impossible to distinguish between these two diseases. The different varieties of erythema are much more unlike each other, than many cases of erythema and roseola." 342.

"You may remember that sometimes it is very white; and sometimes black. That will do as well as remembering *vulgaris, alphoides,* and *nigricans.* It is a great mercy that we have no other names given for the intermediate shades." 379.

What will he say to the "dermocaceccrises, dermocryptoncoses, tricho-dermo-atoxapathy," &c. of a recent author, which are almost as bad as the "spondylexarthreticus, and hydrocatarrhophicus" of Plouquet. We ought to be thankful we have not many of these cacophonies.

Further on, likewise, speaking of the "sonorous respiration" in bronchitis, he observes, "some choose to use the Latin word *ronchus;* and some say *râle,* and would not say *rattle* for the world, because it is not scientific." Not so much perhaps because it is not "scientific," but because it is not *fashionable.* It would puzzle any but an Englishmen to know why *râle* should be preferable to *rattle; bruit* to *sound; scie* to *saw,* &c. If our language were very meagre and contracted, there would be sufficient excuse for borrowing of our neighbours; as it is, by this absurd deference to the French, we only increase their national vanity, which is capacious enough, Heaven knows; and such conduct is not reciprocated by them, for rather than adopt an English word, if they had not one of their own corresponding to it, they would manufacture one.

We are sorry that we cannot extract largely from the pages devoted to phthisis and diseases of the heart, which are treated of in a style commensurate with their vast importance. Almost every subject exhibits great research and acumen, original and comprehensive views, and an extensive acquaintance with physiology, pathology, and all the known resources of the healing art. Some affections which are scarcely noticed in other works, as glanders, hay-asthma, &c. are also considered; and there is much interesting detail connected with these topics.

In addition to the sterling practical matter in which the work everywhere abounds, we have all the charm of varied and lively illustration, drawn not merely from writings strictly medical, but from the pages of history, poetry, and general literature; so that the casual reader would be surprised to find many parts of the book as entertaining as a novel; for instance, idiocy, insanity, and other topics. The Doctor has not thought it necessary to be crabbed and technical, dry and repulsive. He has evidently striven to render his subject inviting to his auditors; to be "all things" to them, that he might win their affections and attention, and thus inculcate the more successfully the great and important truths he had to convey. We are also happy to bear testimony to the spirit of candour and fairness that the work exhibits; there is occasionally, it is true, a dash of egotism, but it is by no means offensive, and we look in vain for instances of that dogmatism and overweening conceit with which some of Dr. Elliotson's enemies are accustomed to charge him. On the contrary, he appears anxious to do justice to his contemporaries and predecessors, and ingenuously acknowledges when he has failed, as well as when he has succeeded, and what he is ignorant of as well as what he knows.

The style throughout is nervous and masculine; if not elegant, by no means coarse, and always free and perspicuous: and the colloquial form of vivâ voce delivery being retained, there is a character of graceful freedom in every sentence, that we seem to merge the didactic lectures in the familiar friend, speaking to us of his own every-day experience. This circumstance, however, doubtless occasions many of the recapitulations and redundancies we before spoke of: they may be necessary in imparting oral instruction, but are best avoided when the language so repeated has taken the permanent form of "print."

The Editor, Dr. Rogers, has acquitted himself in a very admirable manner, and we cordially assent to all that he claims for himself in the preface. He also deserves the negative commendation of not encumbering the text with multifarious notes: most readers prefer to judge and compare for themselves. He has contributed an Appendix, containing a Synopsis of Cullen's Nosology, extracts from Dr. Elliotson's Elements of Physiology, and copious "excerpta," both here and in the body of the work, from the author's important Treatise on Diseases of the Heart. He has also furnished some ingenious physiological tables by the late Dr. Fletcher. These additions are valuable, but we could have wished to see a selection of good "formulæ" for the various remedies spoken of in the work, similar to those in the excellent publications of Drs. Gregory and Copland. We beg to suggest that such an Appendix be prepared for the next edition, which we expect will be called for at no distant date. It would also be an improvement if the pages were headed with the titles of the different subjects of which they treat. We must likewise remind the Editor that there are several typographical errors; and we have noticed a superabundance of punctuation, chiefly of commas and semicolons; we think we could point out some scores which might be advantageously "deleted." But these are but as maculæ on the face of the sun, and we only mention them that they may be looked to in another edition; some few errors will creep in notwithstanding the most vigilant care. After a diligent perusal we have formed the highest opinion of this edition of Dr. Elliotson's Principles and Practice of Medicine. It is the most modern work on the subject, and is every way calculated to represent to foreigners the present state of practical medicine among the best practitioners of our country. We think it unnecessary to recommend it, because it will recommend itself, and command success by its own intrinsic merits. We have only to add that it forms a goodly volume, containing upwards of 1100 octavo pages, printed in a bold and clear type, and is published at a very moderate price.

